# Artificial intelligence in ultrasound-guided regional anesthesia: A scoping review

**DOI:** 10.3389/fmed.2022.994805

**Published:** 2022-10-25

**Authors:** Dmitriy Viderman, Mukhit Dossov, Serik Seitenov, Min-Ho Lee

**Affiliations:** ^1^Department of Biomedical Sciences, Nazarbayev University School of Medicine, Nur-Sultan, Kazakhstan; ^2^Department of Anesthesiology and Critical Care, Presidential Hospital, Nur-Sultan, Kazakhstan; ^3^Department of Computer Sciences, Nazarbayev University School of Engineering and Digital Sciences, Nur-Sultan, Kazakhstan

**Keywords:** artificial intelligence, ultrasound, regional anesthesia, ultrasound-guided regional anesthesia, training, machine learning, peripheral nerve block, sono-anatomy

## Abstract

**Background:**

Regional anesthesia is increasingly used in acute postoperative pain management. Ultrasound has been used to facilitate the performance of the regional block, increase the percentage of successfully performed procedures and reduce the complication rate. Artificial intelligence (AI) has been studied in many medical disciplines with achieving high success, especially in radiology. The purpose of this review was to review the evidence on the application of artificial intelligence for optimization and interpretation of the sonographic image, and visualization of needle advancement and injection of local anesthetic.

**Methods:**

To conduct this scoping review, we followed the PRISMA-S guidelines. We included studies if they met the following criteria: (1) Application of Artificial intelligence-assisted in ultrasound-guided regional anesthesia; (2) Any human subject (of any age), object (manikin), or animal; (3) Study design: prospective, retrospective, RCTs; (4) Any method of regional anesthesia (epidural, spinal anesthesia, peripheral nerves); (5) Any anatomical localization of regional anesthesia (any nerve or plexus) (6) Any methods of artificial intelligence; (7) Settings: Any healthcare settings (Medical centers, hospitals, clinics, laboratories.

**Results:**

The systematic searches identified 78 citations. After the removal of the duplicates, 19 full-text articles were assessed; and 15 studies were eligible for inclusion in the review.

**Conclusions:**

AI solutions might be useful in anatomical landmark identification, reducing or even avoiding possible complications. AI-guided solutions can improve the optimization and interpretation of the sonographic image, visualization of needle advancement, and injection of local anesthetic. AI-guided solutions might improve the training process in UGRA. Although significant progress has been made in the application of AI-guided UGRA, randomized control trials are still missing.

## Background

Regional anesthesia (RA) is increasingly used in pain management for various surgical procedures. Ultrasound (US) has been used to facilitate the performance of the regional block, increase the percentage of successfully performed procedures and reduce the complication rate. US rapidly gained popularity among practitioners due to its portability, absence of radiation, and the ability to track the performance of the procedure in a real-time fashion (1). Other benefits of US in regional anesthesia include direct visualization of nerves, blood vessels, muscles, bones, tendons, faster sensory onset time, visualization of the local anesthetic spread during injection, timely recognition of maldistribution of local anesthetics, possible prevention of complications (e.g., inadvertent intravascular injection, intra-neuronal injection of local anesthetic), longer duration of the block, possible avoidance of painful muscular contractions during nerve stimulation in cases of fractures), possible improvement of quality of block (2–7).

However, the application of ultrasound-guided regional anesthesia is associated with several technical challenges, which are especially prevalent in trainees and not experienced clinicians. The performance of a block can be complicated by the loss of the reflective signal between the needle and probe, which decreases the needle visibility, especially if a deep block is performed or a patient is overweight. Moreover, bone or hyperechoic soft tissue along the needle trajectory may worsen needle visibility. Therefore, clear needle localization is challenging, especially if deep blocks are performed.

Artificial intelligence (AI) has been studied in many medical disciplines with achieving high success, especially in radiology (8). Since sonographic visualization is commonly used in regional anesthesia, AI solutions might be useful for practitioners in anatomical landmark identification and reducing or avoiding possible complications such as injury to the nerve, artery, vein, and puncture of the peritoneum, pleura, internal organs, as well as local anesthetic systemic toxicity. AI-guided solutions can improve the optimization and interpretation of the sonographic image, and visualization of needle advancement and injection of local anesthetic (3–7).

The purpose of this scoping review (SR) was to synthesize and analyze the evidence on the application of artificial intelligence for optimization and interpretation of the sonographic image, and visualization of needle advancement and injection of local anesthetic.

## Methods

### Protocol

To conduct this SR, we followed the PRISMA guidelines during the design, implementation, and reporting of this review.

We followed the PICO items:

P (patient population): 1. Age 18 years of age and older;

I (intervention): Artificial intelligence-assisted in ultrasound-guided regional anesthesia.

C (comparator): standard methods.

Participants/population: Patients undergoing surgery under regional anesthesia.

### Goals of the SR

1. To review and assess the value and performance of AI-assisted UGRA in different anatomical regions and nerves;
2. Machine learning models and algorithms;
3. To assess the benefits of automatic target detection;
4. To assess risks, failures and limitations of the AI-assisted UGRA.

### Inclusion criteria

1. Application of Artificial intelligence-assisted in ultrasound-guided regional anesthesia;
2. Any human subject (of any age), object (manikin), or animal.
3. Study design: prospective, retrospective, RCTs;
4. Any method of regional anesthesia (epidural, spinal anesthesia, peripheral nerves);
5. Any anatomical localization of regional anesthesia (any nerve or plexus).
6. Any methods of artificial intelligence;
7. Settings: Any healthcare settings (Medical centers, hospitals, clinics, laboratories).

### Exclusion criteria

1. Not enough data reported;
2. Out of inclusion criteria;
3. Application of AI other than anatomic landmark identification and guidance in UGRA (e.g., for AI-based prediction of the need for nerve blocks, AI for robotic nerve blocks, prediction of response of regional anesthesia.

## Literature search

### Search strategy

Studies were identified by electronic search in PubMed, Google Scholar, Embase, using the following search terms “Artificial intelligence,” “Deep learning,” “Ultrasound,” “Ultrasound-guided,” “Needle identification,” “Needle tracking,” “Regional anesthesia,” “Peripheral nerve block.” Additionally, we performed a manual search of the articles using the references from the published studies. Publications in English, German and Russian languages were considered.

### Data collection and extraction

The data were extracted into a standardized form. Two authors independently screened the titles and abstracts for eligibility. The following data were extracted: citation, author, year, gender, study goals, sample size, types of surgery, nerve block, the algorithm of AI, comparator, the purpose of AI, benefits, risks and limitations of the study, model performance data and conclusions.

## Results

The systematic searches identified 78 citations. After the removal of the duplicates, 19 full-text articles were assessed; and 15 studies were eligible for inclusion in the review (Supplementary Figure 1). The studies were conducted on healthy subjects, parturients in labor or scheduled for cesarean delivery, bovine/porcine lumbosacral, and bovine/porcine lumbosacral spine phantoms.

### Characteristics of study goals

The included studies aimed to assess the value of AI by the following methods:

- Studying nerve structure and ultrasound image tracking (9);
- Assessing deep-learning performance for nerve tracking in ultrasound images (10);
- Studying the accuracy of real-time (AI) -based anatomical identification (11);
- Assessment of CNN-based framework for needle detection in curvilinear 2D US (12);
- Evaluation of success rate of spinal anesthesia of AI-assisted methods (13);
- Using AI for precise needle target localization (14);
- Identification of musculocutaneous, median, ulnar, and radial nerve) and blood vessels (15);
- Assessment of the utility of ScanNav to identify structures, teaching and learning UGRA, and increase operator confidence (16);
- Assessment of UGRA expert perception of risks of the use of ScanNav (risk of block failure, unwanted needle trauma (eg, arteries, nerves, and pleura/peritoneum (16);
- Identification of the difference in accuracy between deep learning (DL)-powered ultrasound guidance and regular ultrasound images; the use of artificial intelligence to optimize regional anesthesia puncture path; to identify the effectiveness of ultrasound-guided imaging “scapular nerve block” surgical pain of the fracture (17).

### Anatomical region and the nerves

It was found that AI-assisted UGRA has the potential to facilitate the identification of anatomical structures and assist non-experts in locating the correct ultrasound anatomy to perform the intervention. The previous reports highlighted the apparent deficiencies in anatomical knowledge among junior anesthesiologists (18). These deficiencies may be supported by the assistance of ultrasound image interpretation. Therefore, such assistive AI approaches could improve the probability of successful interventions and reduce their risks (18).

Thus, artificial intelligence-assisted ultrasound-guided target identification was used for the identification of the following anatomical structures (nerves): musculocutaneous, median, ulnar, and radial nerves, “interscalene-supraclavicular” and “infraclavicular brachial plexus,” “axillary level brachial plexus,” “erector spinae plane,” rectus sheath, “suprainguinal fascia iliaca,” adductor canal, “popliteal sciatic nerve,” “transverses abdominis plane,” anesthesia in the lower vertebrae regions (sacrum, intervertebral gaps, and vertebral bones), sciatic nerves, femoral nerve, subarachnoid and epidural spaces, facet blocks, navigation of blood vessels during UGRA (9–15, 18–21) (Table 1).

**Table 1:** Study and cohort information.

**Author, country, year**	**Study goal**	**Study population (diagnosis)**	**Sample size Gender (males %)**	**Region of body studied**
Bowness et al., 2021 (18)	Assess the AI anatomy identification	Healthy population	244	Interscalene-supraclavicular level brachial plexus block Rectus sheath block Axillary level brachial plexus Erector spinae plane block Suprainguinal fascia iliaca block Adductor canal block Popliteal level sciatic nerve block
Alkhatib et al., 2018, France (9)	To study nerve structure and ultrasound images tracking	–	10 6 (60%) males 4 (40%) females	Median nerve identification
Alkhatib et al., 2019, France (10)	To study the deep-learning performance for nerve tracking in ultrasound images	-	42	Median & sciatic nerves
Gungor et al., 2021 (11)	To study the accuracy of real-time (AI) -based anatomical identification	Healthy population	40 20 (50%) males 20 (50%) females	Block regions: Supraclavicular, infraclavicular, and transversus abdominis plane (TAP)
Hetherington et al., 2017 (19)	Detect the lower vertebral level	–	20	Anesthesia in the lower vertebrae regions (sacrum, intervertebral gaps, and vertebral bones)
Huang et al., 2019 China (20)	femoral nerve on ultrasound images	–	–	Femoral nerve
Mwikirize et al., 2018 (12)	CNN-based framework for needle detection in curvilinear 2D US	bovine/porcine lumbosacral spine phantom	–	
Oh et al., 2019, Singapore (13)	Success rate of spinal anesthesia	Obstetric women	100	Spinal anesthesia
Pesteie et al., 2017 (14)	Precise needle target localization	–	33	–
Smistad et al., 2018, Norway (15)	Identification of musculocutaneous, median, ulnar, and radial nerve) and blood vessels	Healthy volunteers	49	Axillary nerve block: four nerves (musculocutaneous, median, ulnar, and radial nerve) and blood vessels
Tran et al., 2010, Canada (21)	Features of the lumbar anatomy	Parturients in labor or scheduled for cesarean delivery	20	Epidural anesthesia
Bowness et al., 2022 (16)	Assessment of the utility of ScanNav to identify structures, teaching and learning UGRA and increase operator confidence. Assessment of UGRA expert perception of risks of the use of ScanNav (risk of block failure, unwanted needle trauma (eg, arteries, nerves, and pleura/peritoneum	Healthy volunteers	2	Nine peripheral nerve block regions The upper limb (the “interscalene-,” “upper trunk-,” “supraclavicular-,” “axillary-level brachial plexus” regions; “Erector spinae plane block,” “rectus sheath plane block regions”; the “suprainguinal level fascia iliaca plane,” “adductor canal and popliteal-level sciatic nerve blocks.”
Bowness et al., 2022 (22)	Expert-level AI model performance evaluation	Healthy adult subjects	40	**Upper-extremity blocks:** “upper trunk of the brachial plexus,” “interscalene-level brachial plexus,” “supraclavicular-level brachial plexus,” “axillary-level brachial plexus”
				**Thoraco-abdominal blocks**: erector spinae plane, rectus sheath block. Lower-extremity blocks: “suprainguinal fascia iliaca,” “adductor canal and distal femoral triangle,” “popliteal-level sciatic nerve blocks.”
Yang et al., 2022 (23)	Development a deep learning algorithm to locate the “interscalene brachial plexus” based on ultrasound images to aid anaesthesiologists.	Patients	1076 (dataset −11 392 images	Interscalene brachial plexus
Liu et al., 2021 (17)	To identify difference in accuracy between deep learning-powered ultrasound guidance and regular ultrasound images; the use of artificial intelligence to optimize regional anesthesia puncture path; to identify the effectiveness of ultrasound-guided imaging “scapular nerve block” surgical pain of the fracture	Patients	100	“Scapular nerve block”

### Machine learning models and algorithms

The goal of the included studies was to accurately identify the target region (i.e., nerve block) on the ultrasound images in real-time (4). Therefore, some machine-learning methods have been proposed (Table 1) and their key techniques can be divided into (1) anatomic region segmentation, (2) target detection (i.e., feature extraction), and 3) tracking algorithm (9–15, 18–21).

The U-net is a popular DNN framework to find the region of interest by its fast and precise segmentation performance (Table 2).

**Table 2:** Artificial intelligence method and its purpose.

**Study citation, first author**	**Machine learning model**	**Purpose of ML**	**Benefits**	**Risks and limitations**
Bowness et al. (18)	ScanNav Anatomy Peripheral Nerve Block system (Intelligent Ultrasound Ltd [IUL], Cardiff, UK) - deep convolutional neural networks based on the U-Net architecture	To identify anatomical regions	Identifying the specific anatomical structures, correct ultrasound view to anesthetists and standardization of clinical procedure	Model-related: Recognizes only anatomical structures on images
Alkhatib et al. (9)	Adaptive Median Binary Pattern approach Joint Adaptive Median Binary Pattern approach Three tracking algorithms: particles filter, Mean Shift and Kanade-Lucas-Tomasi (KLT) techniques	To imrove tracking procedure	Automatic detection and tracking of nerve structure, ROIs	Model-related: Nerve appearance might be similar to surroundings Difficulties in real-time tracking Risk of error after many iterations
Alkhatib et al. (10)	Deep learning methods: C-COT, ECO, CNT, MDNet SANet, SiameFC, CFNet, DCFNet, MCPF, HDT, HCFT CREST, DLT, PF-AMBP	Median and the sciatic nerves	Good performance Overcoming noise difficulties No need for pre-filtering images	Model-related: Nerve appearance might be similar to surroundings Failure of retracing the nerve
Gungor et al. (11)	Nerveblox, Smart Alfa Teknoloji San	Identify anatomical structures	A real-time interpretation of anatomic structures	Model-related: Low accuracy in pediatric/geriatric patients
Hetherington et al. (19)	SLIDE (Spine Level IDEntification) System based on deep convolutional neural network	transverse spinal ultrasound planes classification	Successful detection of vertebral regions at real-time speed	Model-related: Failure in identifying the difference between gap and bone images Real-time speed considerations
Huang et al. (20)	Deep learning model: U-Net	identify femoral nerve	Fast training and forecasting of the method Real-time segmentation	Study-related: Small sample size Limited number of images No data augmentation
Mwikirize et al. (12)	Deep learning (DL) based on convolution neural networks (CNNs)	Evaluate the new method	2D US data; deep convolution neural network usage detection data and intensity invariant feature maps	Model-related: Cannot systematically find the needle Relying on an expert sonographer
Oh et al. (13)	to detect the inter-spinous images	Localize L3/4	Confirm the sonographic images and structures. Time saving method Less possible complications	Study-related: Lack of a comparator arm Highly specific algorithm. The system is validated by current study population Absence of complex spinal anatomy, obesity, pediatric and geriatric patients. The risk of misinterpretation of fusion or reduced interspinous distance
Pesteie et al. (14)	CNN-based machine learning technique	Evaluate the convolutional network architecture	Few outliers in detecting the needles Performance is better compared with others	Model-related: Not running in real time
Smistad et al. (15)	Deep convolutional neural network – U-Net	Identify musculocutaneous, median, ulnar, and radial nerves and blood vessels	Accurate detection of blood vessels, median and ulnar nerves Real-time identification Direct comparison of 4 methods	Study-related: Small sample size Low precision and recall values Poor identification of musculocutaneous, radial nerves
Tran et al. (21)	MATLAB algorithm	Detect the LF depth	Helps to find the epidural space and measure the skin-to-LF depth An implementation in a wide range of ultrasound machines.	Model-related: Insignificant errors and failures to detect the LF mean Poor image quality might result in unsatisfactory outcomes
Bowness et al. (16)	ML/DL	Identification of the anatomical structures	Potential to support non-experts in training /clinical practice, as well as experts in teaching UGRA. It may promote the uptake and spread of UGRA.	Model-related: Experts reported an increase in risk
Bowness et al. (22)	DL (based on U-Net architecture)	Identification of the anatomical structures; highlighting anatomical structures of interest	High TP/TN and low FP/FN rates in key anatomical structure identification	Model-related: UGRA itself has not reduced the incidence of nerve injury; Study-related: remote expert were not present when the subjects were scanned.
Yang et al. (23)	DL		The developed model located the “interscalene brachial plexus” more accurately compared to nonexperts.	
Liu et al. (17)	DL, SegNet Model	to optimize regional anesthesia puncture path;	DL ultrasound guided imaging for scapular nerve block in scapular fracture surgery was more efficient, significantly shortened the time of performing nerve block and reduced complications compared to traditional method.	

ML, machine learning; PPV, positive predictive value; NPV, Negative predictive value; AUC-area under the curve; FP-false-positive; FN-false-negative.

The feature extraction methods were divided into typical hand-crafted features and CNN approaches. In general, the hand-crafted feature is more suitable for the smaller size dataset, while the CNN has the strength for more complex classification problems with an automatic feature extraction in the end-to-end framework. The SIFT, LBP, AMBP, HOG, and bag-of-features are well-known hand-craft features and have shown promising results in the US images (9, 21, 24).

The deep-learning models are less optimized with the time complexity, and they predict the given sequential input image independently. Therefore, the model performance is highly sensitive to nerve disappearance due to artifact noise, illumination, or occlusion. Tracking algorithms are one solution for not losing the target object (i.e., nerve) from the initially represented features in the ROI. Previous studies have shown an efficient tracking performance with the conventional MI algorithms, such as Kalman/particle filter (25), mean shift (26), kanade-Lucas-Tomasi (KLT), etc (8). The DNN-based tracking approaches have recently been proposed in the CV domain, however, it is rarely used in sonographic image. Alkhatiba et al. (10) firstly investigated the performance of 13 DNN models, (e.g., ECO, SANet, SiameFC, CFNet) and compared their performance with the hand-crafted feature (AMBP-PF). The study indicates that the CNN models have outperformed the traditional MI algorithms in terms of accuracy and stability, and reported some important findings for enhancing the performance by (1) using a deeper layer, (2) reducing the redundancies, (3) incorporating particle filter (or RNN) in the network.

In many cases, DNN approaches have been implemented along with data augmentation, knowledge transfer, and visualization to overcome the limitations, i.e., small-size datasets, parameter optimization, and low interpretability, respectively. Positional augmentations (scaling, affine transformation, etc.) are common techniques; Pesteie et al. (14) proposed Walsh-Hadamard transform to train a deep network with a set of distinctive directional features from the spatial domain. Mwikirize et al. (12) employed transfer learning, where the network weights are initialized by non-medical images, then fine-tuned with US images.

Overall performance of detection rate were between 88 and 95% and 0.638–0.722 in terms of the precision rates, and IoU evaluation, respectively (19, 20), and tracking performance was above 85% (10).

### Benefits of automatic target detection

The main benefits included an automatic detection and tracking of nerve structure, overall good performance, assistance in successful recognition of specific anatomical structures, confirming the correct placement of the needle, ultrasound view to anesthetists and standardization of clinical procedure, a real-time interpretation of anatomic structures for immediate decision-making during blocks, provides automatized nerve block using the remote control system, successful detection of vertebral regions at the real-time speed (9–15, 18–21, 26, 27). It was reported that artificial intelligence can provide assistance for both novice trainees and experienced clinicians unfamiliar with ultrasound techniques. The ultrasound-guided approach does not increase as the automated ultrasound-guided neuraxial technique takes less than a minute. The automated approach was reported to result in a high rate of first attempt success rate that could reduce the complications from multiple entry attempts (19, 25–28). In another study, DL-assisted ultrasound-guided imaging for scapular nerve block in scapular fracture surgery was more efficient, significantly shortened the time of performing nerve block, and reduced complication rate compared to the traditional method (17).

### Risks, failures, and limitations of the AI-assisted UGRA

Although the application of automated solutions has several benefits, the risks, failures, and limitations were also reported. Thus, the most important limitation was detection and tracking failure (if the nerve appearance is similar to surrounding areas), risk of the nerve disappearance and identical appearance with the surrounding areas- losing the nerve, issues with real-time tracking error after numerous iterations risk of failing to re-track lost nerve (9–15, 18–21). Another limitation of this technology is the failure of distinguishing osseous images. Although real-time allows proper scanning of block regions, it does not always result in the detection of the whole needle, which can occur at a steep insertion angle. The evidence on the application of AI-assisted technologies in regional anesthesia is still in its initial stage. Thus, limited evidence on accuracy in many patient populations, such as in pediatric/geriatric patients is currently available. Overreliance on an expert sonographer to detect the ground-truth tip localization is a limitation especially if the tip is completely invisible. The algorithm is highly specific only if all landmarks are detected. AI algorithms are not designed or validated in the case of complex spinal anatomy, geriatric patients, obesity patients, and pediatric patients. The risk of image misinterpretation could be high in case of abnormal anatomy (e.g., fusion or reduced interspinous distance).

The following risks were assessed and reported in the studies:

- increased risk of block failure;
- risk of needle trauma to structures (eg, arteries, nerves, pleura, peritoneum);

The assessed complications included:

- nerve injury and “postoperative neurological manifestations”;
- “local anesthetic systemic toxicity”;
- pleural injury (pneumothorax);
- peritoneal injury.

## Discussion

Artificial intelligence-assisted medical image interpretation is one particularly popular research direction in healthcare artificial intelligence (18). Artificial intelligence has been used for the detection of the optimal needle insertion site, estimation of the trajectory of the needle insertion, and facilitating automatic tip localization. Tracking is one of the most widely used tasks in computer vision with such applications as video medical imaging, compression, and robotics.

Several artificial intelligence models have been reported to improve the quality of monographic anatomical target detection. Thus, a multiple model data association tracker has been used to track the left ventricle in the cardiac examination (8).

AI was reported to be helpful in 99.7% of the cases. Identification of specific anatomical structures by ultrasound and confirming the correct view are essential components of ultrasound-guided regional anesthesia (18).

A recent study reported a statistically significant difference between the performances of blocks in different regions. Thus, the rectus sheath and interscalene supraclavicular level brachial plexus regions yielded the lowest results, whereas the adductor canal block and axillary brachial plexus yielded the highest results (18). It is noteworthy to note that two of the three lowest-ranked blocks were plane blocks and anatomical regions that did not have major vascular landmarks in close proximity. Conversely, the highest-ranked anatomic regions have bones and vessels.

The results demonstrate the potential for the clinical utility of AI in UGRA and especially for non-experts users (18). It is challenging to develop the AI algorithms to identify all anatomical features using ultrasound *de novo* due to the diversity, complexity, and operator dependence, such as inter-and intra-individual variation (25). Therefore, automated image interpretation technologies can be trained to identify a wide variety of structures using machine learning (25). This technology could be used to improve the interpretation of ultrasound anatomy by improving target identification such as peripheral nerves and fascial planes, and the mapping of optimal insertion site by detecting the relevant landmarks and guidance structures (such as muscles and bones). The safety profile can be improved by highlighting anatomical structures such as blood vessels) to reduce or even avoid unwanted injury (26).

Although AI-assisted techniques appear to be promising, only a few applications are currently introduced in clinical practice, therefore, the potential for its utilization is yet to be proven (28). Understanding the sonographic anatomy and image interpretation represents critical importance in UGRA. Robust AI-assisted technologies could help clinicians to improve performance and training in ultrasound-guided nerve blocks (26).

AI-assisted technologies can change the practice of UGRA and its education. Anesthesia practitioners should contribute to the transformation of UGRA (28).

Although training can be performed in non-clinical settings, such as educational courses, clinical practice training takes a fundamental role.

AI-assisted UGRA is a novel medical device, with which many clinicians might not be familiar. Therefore, its initial use may be associated with lower confidence, which will improve with time of training and practice.

Generally, the included studies reported a low perception of increased risk associated with using AI assistance, although complications may be clinically important (eg, nerve injury/ LAST). Possible causes of error are related to technological performance, e.g., improper highlighting, which may result misinterpretation of the ultrasound images. Block failure and undesirable trauma to critical structures may be more likely if the practitioner is misleadingly reassured by the color on the screen. Other risks may be related to the usage of the device, e.g., highlighting resulting in distraction or focusing on one object and neglecting another structure.

AI-assisted technology therefore should be used as a source of additional information (image augmentation system) rather than a decision-maker. Furthermore, correct anatomical structure identification can be useful for anesthesiologists, although it does not ensure safe UGRA nor guide needle placement. Therefore, it is the performer's responsibility to take into consideration hazards (26, 28).

### Challenges in using AI regional anesthesia

Tracking anatomical targets in ultrasound-guided procedures can be challenging due to Illumination changes, occlusion, noise, and deformation of the target, which can result in tracking failure. Moreover, the object motion may exhibit abrupt changes; the images may be corrupted by a multiplicative noise leading to false alarms, misdetection; some detected features may not belong to the object. It is important to highlight that the wrongly detected features should be neglected by the tracker because they may mislead medical professional and jeopardize the performance of the procedure (8). Finally, the object shape might change during the tracking (8).

### Barriers to the development of AI-guided UGRA

AI especially CNNs has been improving success in image recognition for many years, since the development of LeNet-5 (29). One of the major reasons for this success is the development of new algorithms, the availability of large data sets, and improvements in hardware (30). The major limitation of training deep CNNs is the requirement of a large number of images; therefore, it is challenging to achieve good results with training deep CNNs using small data sets (24). The challenge, however, can be overcome with transfer learning that can be used for training CNNs on relatively small data sets (24, 27). Transfer learning uses knowledge learned from one area and applies in another area. Transfer learning can solve classification tasks in a new domain using pre-trained CNNs (27). It can be especially useful in medical image classification. To perform image classification, trained CNNs extract features *via* ascending layers of the network (27). CNNs that have been trained on a large number of images have optimized parameters for image recognition, and, therefore, that knowledge can be transferred to use for other tasks. Moreover, only a few products, especially those assessing images in a real-time manner have received regulatory approval.

### Limitations of the current study

The main limitations of this study are that the studies included in this review are small sample size, therefore, the results should be replicated in studies with a larger number of participants with different anatomical abnormalities and comorbidities. Other limitations were an insufficient number of images with a large field of vision and deep depth, no data augmentation limiting image segmentation properties of the studied method. Some studies did not have a comparator arm.

Additional limitation was the “trustworthiness” of clinicians who are under-confident in their anatomical and sonographic expertise, and may over-rely on AI assistance. Therefore, it is important to appreciate that the AI may mistakenly identify the incorrect anatomical location, and a robust understanding of the sonographic anatomy is required even when AI-assisted technologies are used for such procedures (18). Regional anesthesia educators with suitable expertise must be central to training in UGRA and “AI-assisted devices” should not replace expert educators. Trainees should still practice standard methods of sonographic scanning, probe angulation, rotation pressure, and tilt to enhance image acquisition (26).

The next limitation is that the highest were scores demonstrated as regions with major vascular structures and nerves, rather than fascial planes used as a target. Therefore, it is important to find out whether it is due to the operator's input to the system or it is due to the algorithm. This may help to identify what anatomical landmarks and structures are the most beneficial for AI-assisted UGRA (18).

Additionally, the performance of AI-assisted UGRA could be evaluated by diverse criteria such as accuracy, consistency, time complexity, the robustness of noise, and sometimes the visualization results should be qualitatively evaluated by the human. However, current CNN studies have not fully investigated in terms of the model generalization toward a large-size dataset with sufficient evaluation assessments.

### Future development

Ultrasound has become an integral part of regional anesthesia and significantly contributed to its development. Nevertheless, it is challenging to develop excellent skills to interpret ultrasound images and achieve the necessary level of proficiency to perform regional anesthesia safely and reduce the rate of block failure, especially for beginners. Moreover, there is a degree of subjectivity in interpreting ultrasound images, which leads to heterogeneous interpretation even among experienced users. Therefore, the application of AI in UGRA might maximize the benefits of ultrasound guidance, improve efficacy and safety and reduce the failure rate.

Computer vision is one of the most promising areas of application of AI in medicine. Deep learning may hold the highest potential to advance image interpretation in UGRA but a high amount of images would be required for its training, followed by validation prior to its implementation into clinical practice. Therefore, a close collaboration of clinicians and engineers is crucial. Clinicians should play a more active role in these collaborations, since they are instrumental in image acquisition, conducting clinical trials, advising, and overall moving this field forward.

## Conclusion

Since sonographic visualization is commonly used in regional anesthesia, AI solutions might be useful in anatomical landmark identification, reducing or even avoiding possible complications (such as injury to the anatomical structures and local anesthetic systemic toxicity. AI-guided solutions can improve the optimization and interpretation of the sonographic image, visualization of needle advancement, and injection of local anesthetic. AI-guided solutions might improve the training process in UGRA. Although significant progress has been made in the application of AI-guided UGRA, randomized control trials are still missing. More high-quality studies are warranted to generate evidence application of AI-guided UGRA in different patient populations, such as pediatric, and geriatric patients, and in different anatomical regions, nerve blocks, and surgeries. This SR could potentially be used as a basis for future clinical trials and systematic reviews and enable future researchers to identify the directions for applications of AI in regional anesthesia. This review can also enable researchers to avoid the limitations of previous studies, which will be suitable for future systematic reviews and meta-analyses.

## Author contributions

DV: conceptualization, design and methodology, writing initial draft, and editing. MD and SS: data extraction. M-HL: editing and writing. All authors approved the manuscript.

## Funding

This meta-analysis was supported by the Nazarbayev University Faculty Development Competitive Research Grant 2021-2023. Funder Project Reference: 021220FD2851.

## Conflict of interest

The authors declare that the research was conducted in the absence of any commercial or financial relationships that could be construed as a potential conflict of interest.

## Publisher's note

All claims expressed in this article are solely those of the authors and do not necessarily represent those of their affiliated organizations, or those of the publisher, the editors and the reviewers. Any product that may be evaluated in this article, or claim that may be made by its manufacturer, is not guaranteed or endorsed by the publisher.
