# Supplementary material for: Artificial intelligence in ultrasound-guided regional anesthesia: A scoping review
Source: Front Med (Lausanne). 2022 Oct 25;9:994805. doi: 10.3389/fmed.2022.994805 (PMC9640918; doi:10.3389/fmed.2022.994805)
Supplement: Supplementary file 1 [file Data_Sheet_1.docx]

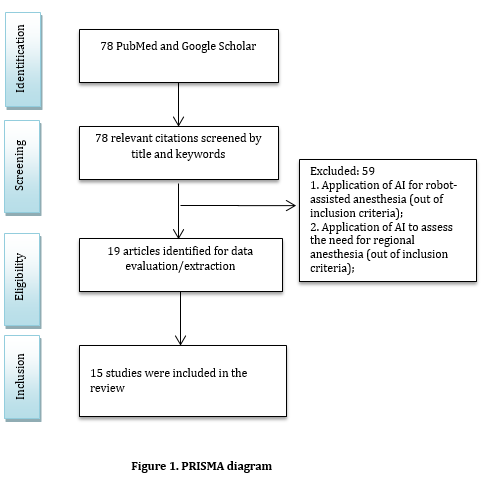


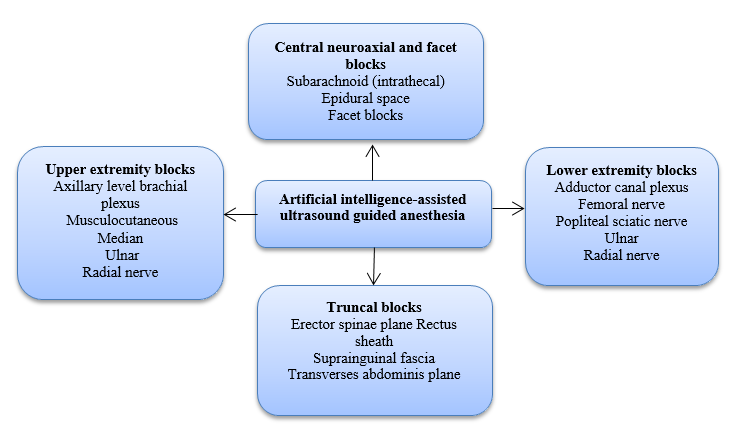


**Figure 2.** Benefits of using artificial intelligence in ultrasound-guided regional anesthesia


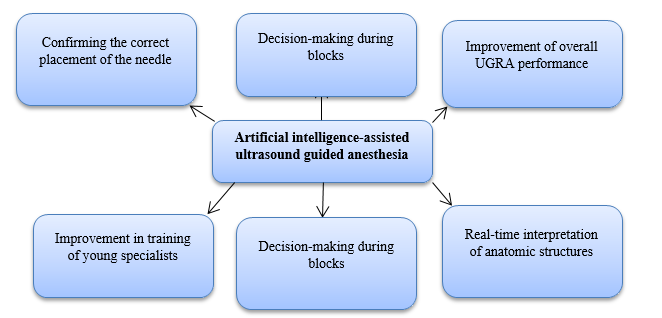


**Figure 3.** Application of artificial intelligence in ultrasound-guided regional anesthesia. This figure depicts nerve block, in which AI-assisted UGRA was studied.
